# 4-(4-Pyrid­yl)pyridinium 3-amino-5-carb­oxy-2,4,6-triiodo­benzoate–5-amino-2,4,6-triiodo­isophthalic acid (1/1)

**DOI:** 10.1107/S1600536810045514

**Published:** 2010-11-13

**Authors:** Kou-Lin Zhang, Guo-Wang Diao, Seik Weng Ng

**Affiliations:** a College of Chemistry and Chemical Engineering, Yangzhou University, Yangzhou 225002, People’s Republic of China; b Department of Chemistry, University of Malaya, 50603 Kuala Lumpur, Malaysia

## Abstract

In the title ammonium carboxyl­ate–carb­oxy­lic acid co-cystal, C_10_H_9_N_2_^+^·C_8_H_3_I_3_NO_4_^−.^C_8_H_4_I_3_NO_4_, the carboxyl­ate anion and carb­oxy­lic acid mol­ecule are linked by O—H⋯O and N—H⋯O hydrogen bonds to form a chain running along the *c* axis of the monoclinic unit cell. The chains are linked by pyridinum and pyridine N—H⋯O hydrogen bonds, generating a layer motif. O—H⋯N and O—H⋯O hydrogen bonds are also observed.

## Related literature

For the crystal structure of 5-amino-2,4,6-triiodo­isophthalic acid monohydrate, see: Beck & Sheldrick (2008 ▶).
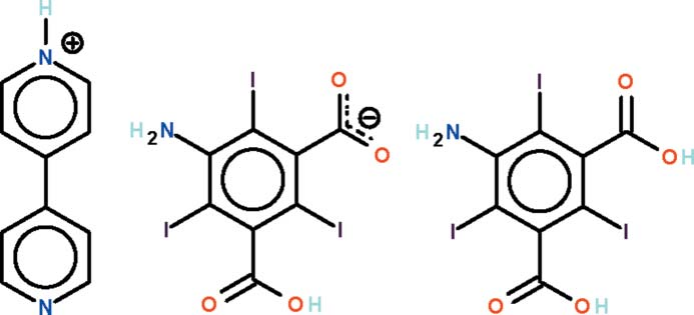


## Experimental

### 

#### Crystal data

C_10_H_9_N_2_^+^·C_8_H_3_I_3_NO_4_^−^·C_8_H_4_I_3_NO_4_
*M* *_r_* = 1273.83
Monoclinic, 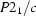

*a* = 7.7388 (4) Å
*b* = 34.2377 (19) Å
*c* = 13.0739 (7) Å
β = 106.506 (1)°
*V* = 3321.3 (3) Å^3^
*Z* = 4
Mo *K*α radiation
μ = 5.66 mm^−1^
*T* = 295 K
0.08 × 0.06 × 0.04 mm

#### Data collection

Bruker SMART APEX diffractometer
Absorption correction: multi-scan (*SADABS*; Sheldrick, 1996 ▶) *T*_min_ = 0.660, *T*_max_ = 0.805
28196 measured reflections
7506 independent reflections
6365 reflections with *I* > 2σ(*I*)
*R*_int_ = 0.037

#### Refinement

*R*[*F* ^2^ > 2σ(*F* ^2^)] = 0.032
*wR*(*F* ^2^) = 0.082
*S* = 1.03
7506 reflections
429 parameters
8 restraints
H atoms treated by a mixture of independent and constrained refinement
Δρ_max_ = 1.25 e Å^−3^
Δρ_min_ = −1.26 e Å^−3^

### 

Data collection: *APEX2* (Bruker, 2007 ▶); cell refinement: *SAINT* (Bruker, 2007 ▶); data reduction: *SAINT*; program(s) used to solve structure: *SHELXS97* (Sheldrick, 2008 ▶); program(s) used to refine structure: *SHELXL97* (Sheldrick, 2008 ▶); molecular graphics: *X-SEED* (Barbour, 2001 ▶); software used to prepare material for publication: *publCIF* (Westrip, 2010 ▶).

## Supplementary Material

Crystal structure: contains datablocks global, I. DOI: 10.1107/S1600536810045514/nk2065sup1.cif

Structure factors: contains datablocks I. DOI: 10.1107/S1600536810045514/nk2065Isup2.hkl

Additional supplementary materials:  crystallographic information; 3D view; checkCIF report

## Figures and Tables

**Table 1:** Hydrogen-bond geometry (Å, °)

*D*—H⋯*A*	*D*—H	H⋯*A*	*D*⋯*A*	*D*—H⋯*A*
N1—H11⋯O8^i^	0.88 (1)	2.22 (4)	2.946 (6)	141 (5)
N3—H3⋯O1	0.88 (1)	1.78 (1)	2.651 (5)	174 (6)
O3—H3o⋯N4^ii^	0.84 (1)	1.75 (2)	2.585 (5)	171 (9)
O5—H5o⋯O1	0.84 (1)	1.77 (3)	2.568 (4)	159 (6)
O7—H7o⋯O2^iii^	0.84 (1)	1.84 (1)	2.679 (5)	174 (7)

Symmetry codes: (i) 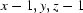; (ii) 
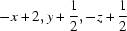
; (iii) 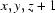.

**Fig. 1.**
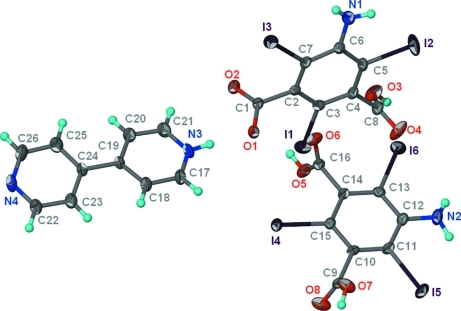
Displacement ellipsoid plot (Barbour, 2001) of C10H9N2+C8H3NO4I3-.C8H4NO4I3 at the 50% probability level; hydrogen atoms are drawn as spheres of arbitrary radius.

**Fig. 2.**
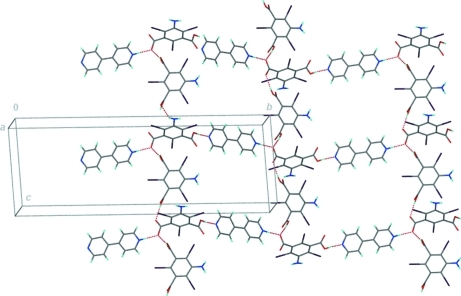
The hydrogen-bonded layer structure. Each amino group forms only one hydrogen bond; the weaker hydrogen bond is are not shown.

## supplementary crystallographic information

### Comment

5-Amino-2,4,6-triiodoisophthalic acid exists as a monohydrated compound for which the acid and water molecules are linked by extensive O–H···O, O–H···N and N–H···O hydrogen bonds to form a three-dimensional network (Beck & Sheldrick, 2008). The acid furnishes a small number of coordination compounds. The attempt to synthesize a cadmium derivative that can be linked by 4,4'-bipyridine gave instead the co-crystal, C_10_H_9_N_2_^+^C_8_H_3_NO_4_I_3_^-.^C_8_H_4_NO_4_I_3_ (Scheme I, Fig. 1).

The carboxylate anion and carboxylic acid are linked by O–H···O and N–H···O hydrogen bonds to form a chain running along the *c*-axis of the monoclinic unit cell. The chains are linked by N_pyridinum_–H···O and N_pyridyl_–H···O hydrogen bonds to generate a layer motif (Fig. 2, Table 1).

### Experimental

An aqueous solution of cadmium dichloride 2.5 hydrate (0.023 g, 0.1 mmol) in water (5 ml) was added to a mixture of 5-amino-2,4,6-triiodoisophthalic acid (0.056 g, 0.1 mmol) in water (5 ml) and sodium hydroxide (0.2 ml, 0.5 *M*). To the solution was added 4,4'-bipyridine (0.016 g, 0.1 mmol) in water (5 ml). The solution was filed; slow evaporation yielded deep yellow crystals were collected (30% yield). CH&N elemental analysis. Calc. for C_26_H_16_I_6_N_4_O_8_: C 24.51, H 1.27, N 4.43%; Found: C, 24.43; H, 1.29; N, 4.50%.

### Refinement

Carbon-bound H-atoms were placed in calculated positions (C—H 0.93 Å) and were included in the refinement in the riding model approximation, with *U*(H) set to 1.2*U*(C).

The amino and acid H-atoms were located in a difference Fourier map, and were refined with distance restraints of N–H 0.88±0.01 and O–H 0.84±0.01 Å; their temperature factors were refined.

The final difference Fourier map had a peak in the vicinity of I5 and a hole in the vicinity of I3.

### Crystal data

**Table:**

C_10_H_9_N_2_^+^·C_8_H_3_I_3_NO_4_^−^·C_8_H_4_I_3_NO_4_	*F*(000) = 2328
*M**_r_* = 1273.83	*D*_x_ = 2.547 Mg m^−^^3^
Monoclinic, *P*2_1_/*c*	Mo *K*α radiation, λ = 0.71073 Å
Hall symbol: -P 2ybc	Cell parameters from 9939 reflections
*a* = 7.7388 (4) Å	θ = 2.4–27.4°
*b* = 34.2377 (19) Å	µ = 5.66 mm^−^^1^
*c* = 13.0739 (7) Å	*T* = 295 K
β = 106.506 (1)°	Prism, yellow
*V* = 3321.3 (3) Å^3^	0.08 × 0.06 × 0.04 mm
*Z* = 4	

### Data collection

**Table:**

Bruker SMART APEX diffractometer	7506 independent reflections
Radiation source: fine-focus sealed tube	6365 reflections with *I* > 2σ(*I*)
graphite	*R*_int_ = 0.037
ω scans	θ_max_ = 27.5°, θ_min_ = 1.7°
Absorption correction: multi-scan (*SADABS*; Sheldrick, 1996)	*h* = −9→9
*T*_min_ = 0.660, *T*_max_ = 0.805	*k* = −44→44
28196 measured reflections	*l* = −16→16

### Refinement

**Table:**

Refinement on *F*^2^	Primary atom site location: structure-invariant direct methods
Least-squares matrix: full	Secondary atom site location: difference Fourier map
*R*[*F*^2^ > 2σ(*F*^2^)] = 0.032	Hydrogen site location: inferred from neighbouring sites
*wR*(*F*^2^) = 0.082	H atoms treated by a mixture of independent and constrained refinement
*S* = 1.03	*w* = 1/[σ^2^(*F*_o_^2^) + (0.0387*P*)^2^ + 6.6421*P*] where *P* = (*F*_o_^2^ + 2*F*_c_^2^)/3
7506 reflections	(Δ/σ)_max_ = 0.001
429 parameters	Δρ_max_ = 1.25 e Å^−^^3^
8 restraints	Δρ_min_ = −1.26 e Å^−^^3^

### Fractional atomic coordinates and isotropic or equivalent isotropic displacement parameters (Å^2^)

**Table:**

	*x*	*y*	*z*	*U*_iso_*/*U*_eq_	
I1	0.89589 (4)	0.633599 (10)	0.26091 (3)	0.03827 (9)	
I2	0.17486 (6)	0.713602 (10)	0.08490 (4)	0.05879 (13)	
I3	0.22245 (5)	0.537807 (9)	0.07851 (3)	0.03820 (9)	
I4	0.63572 (4)	0.521651 (8)	0.65077 (2)	0.03066 (8)	
I5	0.57056 (6)	0.666532 (10)	0.90690 (3)	0.04259 (10)	
I6	0.36084 (5)	0.672374 (10)	0.42509 (3)	0.04230 (10)	
O1	0.6872 (5)	0.54473 (9)	0.2775 (2)	0.0315 (7)	
O2	0.6823 (5)	0.54041 (10)	0.1070 (3)	0.0370 (8)	
O3	0.6689 (6)	0.71617 (11)	0.1148 (3)	0.0430 (9)	
H3O	0.724 (12)	0.7376 (14)	0.130 (7)	0.12 (4)*	
O4	0.6193 (7)	0.72189 (11)	0.2738 (3)	0.0577 (12)	
O5	0.6413 (5)	0.58392 (11)	0.4353 (3)	0.0368 (8)	
H5O	0.628 (8)	0.5720 (16)	0.377 (3)	0.055 (19)*	
O6	0.3505 (5)	0.56703 (11)	0.4066 (3)	0.0432 (9)	
O7	0.5332 (5)	0.56640 (12)	0.9082 (3)	0.0394 (8)	
H7O	0.575 (9)	0.5570 (19)	0.970 (2)	0.07 (2)*	
O8	0.8114 (5)	0.56830 (13)	0.8926 (3)	0.0449 (9)	
N1	0.0653 (6)	0.62476 (12)	0.0256 (4)	0.0362 (9)	
H11	0.004 (7)	0.6029 (9)	0.018 (4)	0.045 (16)*	
H12	−0.001 (7)	0.6455 (11)	0.029 (5)	0.048 (17)*	
N2	0.4185 (7)	0.69823 (12)	0.6687 (4)	0.0423 (11)	
H21	0.461 (8)	0.7106 (16)	0.730 (3)	0.052 (18)*	
H22	0.412 (8)	0.7143 (14)	0.615 (3)	0.048 (17)*	
N3	0.8284 (5)	0.47376 (12)	0.3134 (3)	0.0324 (9)	
H3	0.787 (8)	0.4976 (7)	0.299 (5)	0.055 (18)*	
N4	1.1535 (6)	0.28111 (12)	0.3578 (4)	0.0399 (10)	
C1	0.6421 (6)	0.55702 (12)	0.1815 (3)	0.0255 (9)	
C2	0.5326 (6)	0.59425 (12)	0.1600 (3)	0.0228 (9)	
C3	0.6155 (6)	0.63055 (12)	0.1880 (3)	0.0241 (9)	
C4	0.5151 (6)	0.66494 (12)	0.1677 (3)	0.0258 (9)	
C5	0.3306 (6)	0.66243 (12)	0.1171 (4)	0.0263 (9)	
C6	0.2431 (6)	0.62650 (12)	0.0846 (3)	0.0262 (9)	
C7	0.3500 (6)	0.59272 (12)	0.1111 (3)	0.0248 (9)	
C8	0.6070 (7)	0.70421 (13)	0.1925 (4)	0.0315 (10)	
C9	0.6552 (6)	0.57584 (13)	0.8604 (3)	0.0256 (9)	
C10	0.5792 (6)	0.59878 (13)	0.7573 (3)	0.0230 (9)	
C11	0.5321 (7)	0.63773 (13)	0.7594 (3)	0.0281 (9)	
C12	0.4688 (6)	0.66010 (13)	0.6662 (4)	0.0282 (9)	
C13	0.4532 (6)	0.64080 (13)	0.5687 (3)	0.0256 (9)	
C14	0.5027 (6)	0.60201 (12)	0.5647 (3)	0.0228 (8)	
C15	0.5677 (6)	0.58054 (12)	0.6596 (3)	0.0244 (9)	
C16	0.4885 (6)	0.58222 (13)	0.4591 (3)	0.0267 (9)	
C17	0.8615 (7)	0.45571 (14)	0.4059 (4)	0.0361 (11)	
H17	0.8434	0.4689	0.4641	0.043*	
C18	0.9221 (7)	0.41776 (14)	0.4184 (4)	0.0329 (10)	
H18	0.9461	0.4056	0.4846	0.040*	
C19	0.9471 (6)	0.39770 (12)	0.3306 (4)	0.0258 (9)	
C20	0.9103 (7)	0.41793 (14)	0.2341 (4)	0.0337 (11)	
H20	0.9257	0.4057	0.1737	0.040*	
C21	0.8514 (7)	0.45584 (15)	0.2286 (4)	0.0375 (11)	
H21A	0.8271	0.4692	0.1641	0.045*	
C22	1.1572 (7)	0.30185 (14)	0.4440 (4)	0.0393 (12)	
H22A	1.2063	0.2907	0.5109	0.047*	
C23	1.0905 (7)	0.33952 (14)	0.4377 (4)	0.0381 (12)	
H23	1.0955	0.3532	0.4999	0.046*	
C24	1.0163 (6)	0.35702 (13)	0.3398 (4)	0.0273 (9)	
C25	1.0087 (9)	0.33460 (16)	0.2503 (5)	0.0474 (14)	
H25	0.9564	0.3446	0.1823	0.057*	
C26	1.0804 (9)	0.29694 (16)	0.2632 (5)	0.0505 (15)	
H26	1.0766	0.2823	0.2026	0.061*	

### Atomic displacement parameters (Å^2^)

**Table:**

	*U*^11^	*U*^22^	*U*^33^	*U*^12^	*U*^13^	*U*^23^
I1	0.03160 (17)	0.04546 (19)	0.03463 (18)	−0.00668 (14)	0.00433 (13)	0.00376 (14)
I2	0.0485 (2)	0.02261 (17)	0.1008 (4)	0.00977 (15)	0.0140 (2)	0.00154 (18)
I3	0.0456 (2)	0.02220 (15)	0.03956 (19)	−0.00873 (13)	0.00046 (15)	0.00157 (12)
I4	0.04117 (18)	0.02040 (14)	0.02876 (16)	0.00191 (12)	0.00727 (13)	−0.00067 (11)
I5	0.0665 (2)	0.03696 (18)	0.02578 (17)	0.00401 (16)	0.01550 (16)	−0.00919 (13)
I6	0.0586 (2)	0.03503 (18)	0.02813 (17)	0.00338 (15)	0.00399 (15)	0.00980 (13)
O1	0.048 (2)	0.0260 (16)	0.0220 (16)	0.0110 (14)	0.0115 (14)	0.0039 (12)
O2	0.055 (2)	0.0356 (19)	0.0235 (16)	0.0134 (16)	0.0155 (16)	0.0004 (14)
O3	0.063 (3)	0.034 (2)	0.036 (2)	−0.0227 (18)	0.0215 (18)	−0.0058 (16)
O4	0.106 (4)	0.032 (2)	0.041 (2)	−0.021 (2)	0.031 (2)	−0.0154 (17)
O5	0.048 (2)	0.040 (2)	0.0256 (18)	−0.0024 (16)	0.0162 (16)	−0.0078 (15)
O6	0.047 (2)	0.044 (2)	0.0331 (19)	−0.0068 (17)	0.0031 (17)	−0.0128 (16)
O7	0.0347 (19)	0.054 (2)	0.033 (2)	0.0066 (16)	0.0138 (16)	0.0192 (17)
O8	0.0283 (19)	0.066 (3)	0.037 (2)	0.0010 (17)	0.0044 (15)	0.0109 (18)
N1	0.031 (2)	0.027 (2)	0.044 (2)	−0.0006 (17)	0.0019 (19)	−0.0031 (19)
N2	0.073 (3)	0.020 (2)	0.035 (3)	0.011 (2)	0.017 (2)	0.0015 (18)
N3	0.031 (2)	0.027 (2)	0.040 (2)	0.0103 (17)	0.0100 (18)	0.0067 (17)
N4	0.048 (3)	0.024 (2)	0.052 (3)	0.0102 (18)	0.020 (2)	0.0028 (19)
C1	0.032 (2)	0.0177 (19)	0.026 (2)	−0.0002 (17)	0.0067 (18)	0.0001 (17)
C2	0.035 (2)	0.0186 (19)	0.0145 (19)	0.0001 (17)	0.0065 (17)	0.0022 (15)
C3	0.025 (2)	0.028 (2)	0.020 (2)	−0.0031 (17)	0.0066 (17)	−0.0006 (17)
C4	0.040 (3)	0.0163 (19)	0.023 (2)	−0.0027 (17)	0.0116 (19)	−0.0006 (16)
C5	0.034 (2)	0.0162 (19)	0.030 (2)	0.0028 (17)	0.0112 (19)	0.0021 (17)
C6	0.035 (2)	0.023 (2)	0.022 (2)	0.0023 (18)	0.0102 (18)	0.0008 (17)
C7	0.035 (2)	0.0157 (18)	0.023 (2)	−0.0047 (17)	0.0080 (18)	−0.0014 (16)
C8	0.044 (3)	0.018 (2)	0.033 (2)	−0.0019 (19)	0.011 (2)	−0.0022 (18)
C9	0.033 (3)	0.025 (2)	0.019 (2)	−0.0019 (18)	0.0067 (18)	−0.0033 (16)
C10	0.024 (2)	0.027 (2)	0.018 (2)	−0.0044 (17)	0.0061 (16)	−0.0021 (16)
C11	0.039 (3)	0.025 (2)	0.021 (2)	−0.0028 (19)	0.0092 (19)	−0.0037 (17)
C12	0.034 (3)	0.020 (2)	0.031 (2)	−0.0035 (18)	0.009 (2)	−0.0039 (17)
C13	0.030 (2)	0.027 (2)	0.019 (2)	0.0010 (18)	0.0044 (17)	0.0050 (17)
C14	0.028 (2)	0.0212 (19)	0.021 (2)	−0.0026 (16)	0.0088 (17)	−0.0015 (16)
C15	0.028 (2)	0.020 (2)	0.025 (2)	−0.0013 (16)	0.0073 (18)	−0.0020 (17)
C16	0.034 (3)	0.023 (2)	0.021 (2)	0.0009 (18)	0.0050 (19)	0.0002 (17)
C17	0.041 (3)	0.030 (2)	0.039 (3)	0.011 (2)	0.015 (2)	−0.001 (2)
C18	0.043 (3)	0.026 (2)	0.032 (2)	0.010 (2)	0.015 (2)	0.0045 (19)
C19	0.027 (2)	0.022 (2)	0.029 (2)	0.0044 (17)	0.0088 (18)	0.0023 (17)
C20	0.044 (3)	0.027 (2)	0.033 (3)	0.003 (2)	0.015 (2)	0.0045 (19)
C21	0.046 (3)	0.035 (3)	0.034 (3)	0.007 (2)	0.015 (2)	0.008 (2)
C22	0.049 (3)	0.026 (2)	0.042 (3)	0.008 (2)	0.010 (2)	0.004 (2)
C23	0.050 (3)	0.027 (2)	0.038 (3)	0.007 (2)	0.014 (2)	0.003 (2)
C24	0.026 (2)	0.025 (2)	0.032 (2)	0.0052 (17)	0.0108 (19)	0.0011 (18)
C25	0.064 (4)	0.037 (3)	0.039 (3)	0.015 (3)	0.011 (3)	0.004 (2)
C26	0.078 (4)	0.033 (3)	0.041 (3)	0.016 (3)	0.018 (3)	−0.004 (2)

### Geometric parameters (Å, °)

**Table:**

I1—C3	2.110 (4)	C3—C4	1.394 (6)
I2—C5	2.100 (4)	C4—C5	1.395 (7)
I3—C7	2.110 (4)	C4—C8	1.513 (6)
I4—C15	2.095 (4)	C5—C6	1.410 (6)
I5—C11	2.111 (4)	C6—C7	1.407 (6)
I6—C13	2.108 (4)	C9—C10	1.526 (6)
O1—C1	1.276 (5)	C10—C11	1.385 (6)
O2—C1	1.241 (5)	C10—C15	1.402 (6)
O3—C8	1.307 (6)	C11—C12	1.404 (6)
O3—H3O	0.84 (1)	C12—C13	1.409 (6)
O4—C8	1.203 (6)	C13—C14	1.388 (6)
O5—C16	1.307 (6)	C14—C15	1.406 (6)
O5—H5O	0.84 (1)	C14—C16	1.513 (6)
O6—C16	1.211 (6)	C17—C18	1.375 (6)
O7—C9	1.313 (6)	C17—H17	0.9300
O7—H7O	0.84 (1)	C18—C19	1.398 (6)
O8—C9	1.190 (6)	C18—H18	0.9300
N1—C6	1.374 (6)	C19—C20	1.396 (6)
N1—H11	0.88 (1)	C19—C24	1.485 (6)
N1—H12	0.88 (1)	C20—C21	1.371 (7)
N2—C12	1.366 (6)	C20—H20	0.9300
N2—H21	0.88 (1)	C21—H21A	0.9300
N2—H22	0.88 (1)	C22—C23	1.383 (7)
N3—C17	1.316 (6)	C22—H22A	0.9300
N3—C21	1.323 (7)	C23—C24	1.382 (7)
N3—H3	0.878 (10)	C23—H23	0.9300
N4—C26	1.322 (7)	C24—C25	1.387 (7)
N4—C22	1.325 (7)	C25—C26	1.395 (7)
C1—C2	1.512 (6)	C25—H25	0.9300
C2—C7	1.376 (6)	C26—H26	0.9300
C2—C3	1.399 (6)		
			
C8—O3—H3O	111 (6)	N2—C12—C11	122.2 (4)
C16—O5—H5O	108 (4)	N2—C12—C13	121.2 (4)
C9—O7—H7O	115 (5)	C11—C12—C13	116.6 (4)
C6—N1—H11	122 (4)	C14—C13—C12	122.0 (4)
C6—N1—H12	116 (4)	C14—C13—I6	119.0 (3)
H11—N1—H12	113 (6)	C12—C13—I6	119.0 (3)
C12—N2—H21	117 (4)	C13—C14—C15	120.0 (4)
C12—N2—H22	122 (4)	C13—C14—C16	120.9 (4)
H21—N2—H22	110 (6)	C15—C14—C16	119.1 (4)
C17—N3—C21	121.0 (4)	C10—C15—C14	119.0 (4)
C17—N3—H3	127 (4)	C10—C15—I4	121.8 (3)
C21—N3—H3	112 (4)	C14—C15—I4	119.1 (3)
C26—N4—C22	118.5 (4)	O6—C16—O5	126.6 (4)
O2—C1—O1	124.1 (4)	O6—C16—C14	122.2 (4)
O2—C1—C2	119.6 (4)	O5—C16—C14	111.2 (4)
O1—C1—C2	116.3 (4)	N3—C17—C18	121.6 (5)
C7—C2—C3	119.3 (4)	N3—C17—H17	119.2
C7—C2—C1	120.1 (4)	C18—C17—H17	119.2
C3—C2—C1	120.6 (4)	C17—C18—C19	119.2 (4)
C4—C3—C2	120.7 (4)	C17—C18—H18	120.4
C4—C3—I1	119.4 (3)	C19—C18—H18	120.4
C2—C3—I1	119.9 (3)	C20—C19—C18	117.3 (4)
C3—C4—C5	118.6 (4)	C20—C19—C24	121.0 (4)
C3—C4—C8	120.5 (4)	C18—C19—C24	121.7 (4)
C5—C4—C8	120.8 (4)	C21—C20—C19	119.8 (4)
C4—C5—C6	122.4 (4)	C21—C20—H20	120.1
C4—C5—I2	119.7 (3)	C19—C20—H20	120.1
C6—C5—I2	117.9 (3)	N3—C21—C20	121.1 (5)
N1—C6—C7	121.8 (4)	N3—C21—H21A	119.4
N1—C6—C5	121.7 (4)	C20—C21—H21A	119.4
C7—C6—C5	116.4 (4)	N4—C22—C23	122.1 (5)
C2—C7—C6	122.5 (4)	N4—C22—H22A	119.0
C2—C7—I3	119.2 (3)	C23—C22—H22A	119.0
C6—C7—I3	118.3 (3)	C24—C23—C22	120.6 (5)
O4—C8—O3	125.1 (4)	C24—C23—H23	119.7
O4—C8—C4	123.4 (4)	C22—C23—H23	119.7
O3—C8—C4	111.4 (4)	C23—C24—C25	116.6 (4)
O8—C9—O7	125.0 (4)	C23—C24—C19	121.8 (4)
O8—C9—C10	121.5 (4)	C25—C24—C19	121.5 (4)
O7—C9—C10	113.5 (4)	C24—C25—C26	119.3 (5)
C11—C10—C15	119.9 (4)	C24—C25—H25	120.3
C11—C10—C9	121.0 (4)	C26—C25—H25	120.3
C15—C10—C9	119.0 (4)	N4—C26—C25	122.8 (5)
C10—C11—C12	122.5 (4)	N4—C26—H26	118.6
C10—C11—I5	119.9 (3)	C25—C26—H26	118.6
C12—C11—I5	117.5 (3)		
			
O2—C1—C2—C7	76.5 (6)	C10—C11—C12—C13	0.6 (7)
O1—C1—C2—C7	−103.4 (5)	I5—C11—C12—C13	176.7 (3)
O2—C1—C2—C3	−102.6 (5)	N2—C12—C13—C14	−179.6 (5)
O1—C1—C2—C3	77.5 (5)	C11—C12—C13—C14	−1.9 (7)
C7—C2—C3—C4	0.5 (6)	N2—C12—C13—I6	2.7 (6)
C1—C2—C3—C4	179.6 (4)	C11—C12—C13—I6	−179.6 (3)
C7—C2—C3—I1	−179.1 (3)	C12—C13—C14—C15	1.2 (7)
C1—C2—C3—I1	0.0 (5)	I6—C13—C14—C15	178.9 (3)
C2—C3—C4—C5	−1.2 (6)	C12—C13—C14—C16	−178.6 (4)
I1—C3—C4—C5	178.4 (3)	I6—C13—C14—C16	−1.0 (6)
C2—C3—C4—C8	−176.6 (4)	C11—C10—C15—C14	−2.0 (6)
I1—C3—C4—C8	3.0 (5)	C9—C10—C15—C14	−178.1 (4)
C3—C4—C5—C6	−0.8 (7)	C11—C10—C15—I4	−179.7 (3)
C8—C4—C5—C6	174.5 (4)	C9—C10—C15—I4	4.2 (6)
C3—C4—C5—I2	−179.9 (3)	C13—C14—C15—C10	0.8 (6)
C8—C4—C5—I2	−4.5 (6)	C16—C14—C15—C10	−179.4 (4)
C4—C5—C6—N1	−173.5 (4)	C13—C14—C15—I4	178.6 (3)
I2—C5—C6—N1	5.6 (6)	C16—C14—C15—I4	−1.6 (5)
C4—C5—C6—C7	3.3 (6)	C13—C14—C16—O6	−86.0 (6)
I2—C5—C6—C7	−177.6 (3)	C15—C14—C16—O6	94.1 (6)
C3—C2—C7—C6	2.2 (6)	C13—C14—C16—O5	94.3 (5)
C1—C2—C7—C6	−176.9 (4)	C15—C14—C16—O5	−85.6 (5)
C3—C2—C7—I3	−175.9 (3)	C21—N3—C17—C18	0.5 (8)
C1—C2—C7—I3	5.1 (5)	N3—C17—C18—C19	−0.8 (8)
N1—C6—C7—C2	172.8 (4)	C17—C18—C19—C20	0.7 (7)
C5—C6—C7—C2	−4.0 (6)	C17—C18—C19—C24	178.6 (5)
N1—C6—C7—I3	−9.1 (6)	C18—C19—C20—C21	−0.3 (7)
C5—C6—C7—I3	174.0 (3)	C24—C19—C20—C21	−178.2 (5)
C3—C4—C8—O4	−96.2 (6)	C17—N3—C21—C20	0.0 (8)
C5—C4—C8—O4	88.5 (7)	C19—C20—C21—N3	0.0 (8)
C3—C4—C8—O3	84.2 (5)	C26—N4—C22—C23	−1.3 (9)
C5—C4—C8—O3	−91.1 (5)	N4—C22—C23—C24	0.1 (9)
O8—C9—C10—C11	−104.2 (6)	C22—C23—C24—C25	1.6 (8)
O7—C9—C10—C11	74.8 (5)	C22—C23—C24—C19	−178.7 (5)
O8—C9—C10—C15	71.9 (6)	C20—C19—C24—C23	165.6 (5)
O7—C9—C10—C15	−109.2 (5)	C18—C19—C24—C23	−12.2 (7)
C15—C10—C11—C12	1.3 (7)	C20—C19—C24—C25	−14.8 (7)
C9—C10—C11—C12	177.3 (4)	C18—C19—C24—C25	167.4 (5)
C15—C10—C11—I5	−174.7 (3)	C23—C24—C25—C26	−2.2 (9)
C9—C10—C11—I5	1.3 (6)	C19—C24—C25—C26	178.1 (5)
C10—C11—C12—N2	178.4 (5)	C22—N4—C26—C25	0.6 (9)
I5—C11—C12—N2	−5.5 (6)	C24—C25—C26—N4	1.2 (10)

### Hydrogen-bond geometry (Å, °)

**Table:**

*D*—H···*A*	*D*—H	H···*A*	*D*···*A*	*D*—H···*A*
N1—H11···O8^i^	0.88 (1)	2.22 (4)	2.946 (6)	141 (5)
N3—H3···O1	0.88 (1)	1.78 (1)	2.651 (5)	174 (6)
O3—H3o···N4^ii^	0.84 (1)	1.75 (2)	2.585 (5)	171 (9)
O5—H5o···O1	0.84 (1)	1.77 (3)	2.568 (4)	159 (6)
O7—H7o···O2^iii^	0.84 (1)	1.84 (1)	2.679 (5)	174 (7)

Symmetry codes: (i) *x*−1, *y*, *z*−1; (ii) −*x*+2, *y*+1/2, −*z*+1/2; (iii) *x*, *y*, *z*+1.
